# A Conceptual Framework for Organizational Readiness to Implement Nutrition and Physical Activity Programs in Early Childhood Education Settings

**DOI:** 10.5888/pcd11.140166

**Published:** 2014-10-30

**Authors:** Shreela V. Sharma, Mudita Upadhyaya, Daniel J. Schober, Courtney Byrd-Williams

**Affiliations:** Author Affiliations: Mudita Upadhyaya, Courtney Byrd-Williams, Michael and Susan Dell Center for Healthy Living, The University of Texas, School of Public Health, Houston, Texas; Daniel J. Schober, Gretchen Swanson Center for Nutrition and University of Nebraska Medical Center, Omaha, Nebraska.

## Abstract

Across multiple sectors, organizational readiness predicts the success of program implementation. However, the factors influencing readiness of early childhood education (ECE) organizations for implementation of new nutrition and physical activity programs is poorly understood. This study presents a new conceptual framework to measure organizational readiness to implement nutrition and physical activity programs in ECE centers serving children aged 0 to 5 years. The framework was validated for consensus on relevance and generalizability by conducting focus groups; the participants were managers (16 directors and 2 assistant directors) of ECE centers. The framework theorizes that it is necessary to have “collective readiness,” which takes into account such factors as resources, organizational operations, work culture, and the collective attitudes, motivation, beliefs, and intentions of ECE staff. Results of the focus groups demonstrated consensus on the relevance of proposed constructs across ECE settings. Including readiness measures during program planning and evaluation could inform implementation of ECE programs targeting nutrition and physical activity behaviors.

## Introduction

Nearly 11 million children in the United States under the age of 5 are enrolled in some form of nonparental child care during the week (1). Given the high prevalence of obesity and unhealthy eating and physical activity behaviors among these preschool-aged children (2), there is a need for programs promoting healthy behaviors. At early childhood education (ECE) centers, these programs are usually led by the teaching staff. However, teachers at these centers may not have formal training in nutrition or physical activity and may lack knowledge in these areas (3,4). Other challenges may include lack of prioritization of such a program, lack of policy support at the organizational level, and lack of financial resources for staff training, support, and materials (5,6). Furthermore, ECE organizations are unique: they support infants and children aged 0 to 5 years, who depend on their providers to meet their developmental, nutritional, and physical activity needs and who grow faster than children of any other age.

Across multiple sectors (corporate, education, health care, government), organizational readiness is a well-established predictor of successful change and implementation of new programs (7,8). To successfully implement nutrition and physical activity programs in ECE settings, it may be important to first evaluate the readiness of the organizations and their staffs. Such an evaluation allows for early identification and alleviation of potential barriers to implementation. It is especially important in ECE settings because, although federally funded programs such as Head Start have requirements for nutrition and physical activity education (9–11), requirements among centers that are not federally funded vary significantly (12,13). Furthermore, readiness can be program-specific and should be considered as such. An ECE center could have a high level of readiness for a program promoting academic preparation but have a low level of readiness for a program promoting child health, for a multitude of reasons. To our knowledge, no conceptual framework measures organizational readiness to implement nutrition and physical activity programs in ECE settings. The proposed conceptual framework outlines the constructs for organizational readiness and lays the groundwork for developing measures of readiness for implementing these programs. Planning for implementation of nutrition and physical activity programs should build on the knowledge of what resources are currently available to the ECE staff and on the attributes of the staff and administration that will be engaged in program implementation. The objective of this article is to present a validated conceptual framework for organizational readiness to implement nutrition and physical activity programs in ECE centers that serve families with children aged 0 to 5 years.

## Conceptualizing Organizational Readiness to Implement Nutrition and Physical Activity Programs in ECE Settings

Studies in other sectors demonstrate that measuring organizational readiness is critical to successful implementation of new programs (7,14). Organizational readiness and related constructs have been well-defined in these other sectors (7,8,14–17). To develop our conceptual framework, we reviewed the scientific literature in multiple sectors, and we validated our framework through data collected in focus groups conducted among ECE center staff members.

### Development of the conceptual model

ECE centers typically have a 2-level hierarchy: one level consists of management, including a director and other management staff, and the second level consists of teaching staff. Some organizations, such as Head Start, may have a third level, a central administration that manages all centers. This difference in structure could have strong implications for readiness and was a factor in developing our model. Drawing on recent systematic reviews (7,14) and research on organizational readiness (17), we defined readiness for organizational change in ECE settings in the context of “collective readiness.” For any change to be adopted and implemented successfully in an organization, it is necessary to have collective readiness at the individual and organizational levels (14). In the context of ECE organizations, collective readiness describes how well the organization is prepared in terms of resources, organizational operations, and work culture to implement changes in nutrition and physical activity and how supportive an organization is in terms of the collective attitudes, beliefs, and intentions of the ECE staff (teachers and director) to adopt and sustain changes in nutrition and physical activity.

Our conceptual framework (Figure) outlines the 3 main antecedents for readiness that are linked to the successful implementation of new programs (8,14,15). These 3 factors are structural and external factors, staff attributes, and other psychological factors. The structural and external factors are operationalized at the organizational level only, whereas staff attributes and other psychological factors are attitudinal constructs operationalized at both the organizational level (ie, the ECE director’s perception of staff attributes) and the individual level (ie, staff members’ perceptions of their own attributes). Organizational factors also influence individual factors, and all these factors are theorized to collectively inform organizational readiness, which in turn influences program implementation. 

**Figure:**
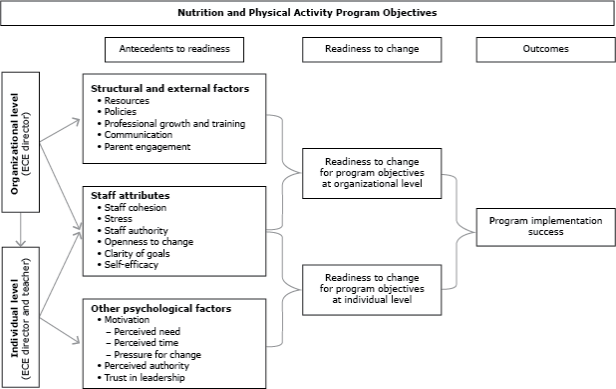
Conceptual framework for organizational readiness to implement nutrition and physical activity programs in early childhood education (ECE) settings.

#### Structural and external factors

Structural and external factors are constructs measured at the organizational level that influence day-to-day functioning and operations of any organization and the organization’s readiness to change (7,18,19). “Resources” are financial, infrastructural, and human resources, including the teaching and administrative staff members who would be available for the nutrition and physical activity program. “Policies” refers to the presence or absence of written organizational policies on behavioral objectives and their alignment with ECE accreditation and licensing requirements. “Professional growth and training” are opportunities for ECE staff and teachers to learn about behavioral objectives, and “communication” refers to the general organizational communication mechanisms and strategies to disseminate health information to staff and families. “Parent engagement” refers to the practices of the organization to engage parents (and families) in implementation of new programs; this construct was added to the framework after the focus groups were conducted. All these constructs are desirable for the adoption and implementation of organizational change and directly influence individual-level factors.

#### Staff attributes

Staff attributes are defined as attitudinal constructs that influence organizational change for nutrition and physical activity program implementation at the individual and organizational levels (7,15,16,20). Staff attributes are measured at the organizational level by the center director and at the individual level by the center director and teaching staff. “Staff cohesion” refers to interconnections and network of relationships in an organization and how well the staff members or teams work with each other. “Stress” is operationalized as physical and mental strain and burnout among organizational members. Stress may lead to staff resistance, which may adversely affect implementation of new programs. Stress can be caused by internal factors (eg, work load) or external factors (eg, personal, medical) and is measured as a combination of these factors. “Staff authority” is a measure of autonomy and flexibility given to staff by higher management to implement new ideas and changes. “Openness to change”’ reflects the general attitude and willingness of the organization and staff to adopt the new program. “Clarity of goals” indicates how well the organization and staff perceive the objectives of the new program as fitting in with the organization’s overall objectives. “Self-efficacy” measures capacity, capability, and confidence of the organization and staff to implement the new program objectives.

#### Other psychological factors

One study defined psychological factors as the beliefs and attitudes of individuals that influence their acceptance and support for any organizational change (21). These factors are primarily measured at the individual level, not the organizational level — ie, the teachers who would engage in program implementation. Drawing on the theory of organizational readiness for change (7) and motivation theory (22), “motivation to change” is a summative construct that measures change as the staff member’s perception of need (ie, do they value the change?), the time required to make the change, and pressure to implement the new program. “Trust in leadership” is the extent of confidence among staff members in the decisions and actions of leadership (supervisor or center director) (23). Finally, “perceived authority” is the staff members’ perception of their own authority in decision making in the organization (23).

For each construct, we summarize its definition and provide an example of how it is operationalized in the context of implementing a nutrition and physical activity program in an ECE setting (Table).

**Table:** Definitions and Operationalization of the Antecedent Constructs to Organizational Readiness for Implementing Nutrition and Physical Activity Programs in Early Childhood Education (ECE) Settings

Antecedent Construct	Definition	Example of Operationalizing of Construct
**Structural and external factors**	**Systems-level constructs that influence day-to-day functioning and operations of any organization and that would influence readiness for change.**	—
Resources	Financial, infrastructural, and human resources including teaching and administrative staff available for implementation of the nutrition and physical activity program.	Do you currently have outdoor space in your ECE center for your children to play and be physically active?
Policies	Written organizational standards for nutrition and physical activity.	Does your ECE center currently have a written policy on nutrition education for your children?
Professional growth and training	Professional growth and training opportunities related to nutrition and physical activity available to the staff and teachers.	Have you or your ECE staff received training in the past year for nutrition in early childhood?
Communication	General organizational communication mechanisms and strategies to disseminate information to staff and parents.	Do you or your ECE staff regularly communicate with parents about their child’s eating habits at school?
Parent engagement	Organizational engagement of parents (and families) in implementation of new programs.	Do you or your ECE staff communicate regularly with parents regarding implementation of new programs at your ECE center?
**Staff attributes**	**Attitudinal constructs that influence organizational change. In the current context, these apply to implementation of the nutrition and physical activity program.**	—
Staff cohesion	Interconnections and network of relationships in an organization and relating to how well the staff members or teams work with each other.	Does your ECE staff here always work as a team when implementing new programs?
Stress	Physical and mental strain and burnout among organization members. Stress may lead to staff resistance, which may adversely affect implementation of programs to promote nutrition and physical activity.	Do you think the heavy workload at your ECE center reduces your effective implementation of new programs?
Staff authority	Measure of autonomy and flexibility given to the staff members by higher management to implement new ideas and changes in the organization.	Are ECE staff members given broad authority in implementing new program objectives?
Openness to change	General attitude and willingness of the organization and staff to adopt change in the organization.	Are you willing to try new ideas about nutrition and physical activity for early childhood?
Clarity of goals	Perception of how the new program objectives (of the change) fit within the overall organizational objectives.	Do you understand how the new program fits as part of your organizational objectives?
Self- efficacy	Capacity, capability, and confidence of the organization and staff to implement the new program.	Upon completion of the training, are you confident that you can implement the new program easily in your ECE center for nutrition education for children?
**Other psychological factors**	**Individual’s beliefs and attitudes that mold acceptance and support for any change.**	—
Motivation	Includes perceived need, perceived availability of time required, and pressure to implement the new program	Do you feel that there is a need in your ECE center to implement the new program?
Trust in leadership	Confidence or sureness of the staff in decisions and actions taken by the leadership (supervisor or center director).	Do you readily follow the opinions of the leadership at your center?
Perceived authority	Perception among staff members of their involvement in the decision making in the organization.	Are ECE staff members typically given broad authority in implementing new program objectives?

Abbreviation: —, does not apply.

### Validation of the conceptual model

The conceptual framework was validated by conducting 3 focus groups comprising 18 ECE center management staff members (16 directors and 2 assistant directors) in Houston, Texas. The focus groups had 9 participants from Head Start, 3 participants from Early Head Start, 1 participant from a private for-profit center, and 5 participants from private nonprofit organizations. Of the 18 organizations represented, 5 organizations served children aged 0 to 5 years, 3 organizations served infants 0 to 12 months, one organization served only children aged 3 years, and 8 organizations served children aged 3 to 5 years. The objective of these focus groups was to obtain a consensus on whether the constructs in the conceptual framework addressed questions that were relevant to ECE organizational staff and to determine whether the constructs were generalizable to a variety of ECE settings. Two trained research project staff members conducted the focus groups using a semistructured interview guide. The transcripts were analyzed independently by 2 reviewers using NVIVO version 10 (QSR International). The focus groups were approved by the Committee for Protection of Human Subjects at the University of Texas Health Science Center, and consent was obtained from participants. At the end of each focus group, the facilitators summarized the consensus among participants. For the purpose of this research, consensus was established when the constructs emerged consistently in all focus groups and more than 90% of the participants concurred. A combination of deductive (using pre-existing theoretical constructs) and inductive (using open coding) methods were used to assess the validity of the conceptual framework (24). Results of the focus groups showed a consensus for each construct and showed that the framework fit well within the ECE context. Through inductive analysis, 4 new themes, or “process factors” emerged: parent engagement in program implementation, cultural diversity, community engagement, and children with special needs. Participants felt that these 4 external factors might influence the implementation of a new nutrition, physical activity, or breastfeeding program. However, consensus was reached on only one, parent engagement in program implementation, which was then added to the conceptual framework.

## Discussion

We propose a validated conceptual framework to assess the organizational readiness of ECE centers to successfully implement new nutrition and physical activity programs. This conceptual framework builds on similar frameworks and constructs of organizational readiness that have been used to inform program implementation in other sectors (7,14–17). Our framework is specific to nutrition and physical activity programs in ECE settings and was broadly validated by focus groups conducted among a diverse set of ECE staff members for consensus on relevance and generalizability. This adds to current ECE program implementation and evaluation practice by introducing standards for assessment of organizational readiness to be considered in the implementation planning process. The use of inductive and deductive methods in analyzing focus group data identified constructs that were not found in the literature review. This highlights the importance of using qualitative work to ensure that any theory or framework is relevant and appropriate contextually.

Organizational readiness is critical: program success relies on optimal implementation, and optimal implementation is influenced by organizational readiness (7,8). A nutrition and physical activity program delivered by ECE staff can be successful only when it is implemented by the staff at a high level of fidelity to the program’s intent (25), and professional training is needed to ensure fidelity. A recent study cited difficulties encountered by ECE staff in delivering several physical activity intervention components as reasons for the inability of the study to demonstrate an effect on the physical activity of the preschool-aged participants (25).

Our framework is currently being used to develop and validate an instrument to assess organizational readiness at an ECE center. A validated instrument to assess readiness could help guide the successful implementation of programs that promote nutrition and physical activity. Methodological considerations include the timing of the assessment and the people assessed (8). If the objective of assessing readiness is to predict the success of implementation of a proposed change, then we recommend that readiness is assessed after the decision to adopt the change occurs but before the implementation process begins. However, if the objective of assessing readiness is to use the assessment as a screener to inform and optimize recruitment of ECE settings into a healthy nutrition and physical activity program, then the assessment should take place before the decision to adopt the change occurs.

We present a validated conceptual framework of organizational readiness for implementation of nutrition and physical activity programs in ECE settings. The framework is timely because of the need for programs targeting healthy nutrition and physical activity in ECE settings. If success is to be achieved in implementing a nutrition and physical activity program, organizational readiness for change must be assessed as part of the program implementation and evaluation plan.
